# Epstein–Barr Virus-Positive Primary CNS Lymphoma in a Patient Receiving Mycophenolate Mofetil: Diagnostic and Therapeutic Considerations

**DOI:** 10.3390/v18050485

**Published:** 2026-04-22

**Authors:** Danielle N. Burner, Giselle Y. López, Justin T. Low, Micah A. Luftig

**Affiliations:** 1 Department of Molecular Genetics and Microbiology, Duke University School of Medicine, Durham, NC 27710, USA; danielle.burner@duke.edu; 2 Department of Pathology, Duke University School of Medicine, Durham, NC 27710, USA; giselle.lopez@duke.edu; 3 Department of Neurosurgery, Duke University School of Medicine, Durham, NC 27710, USA; justin.low@duke.edu; 4 Cancer and Stem Cell Biology, Duke-NUS Medical School, 8 College Road, Singapore 169857, Singapore

**Keywords:** primary central nervous system lymphoma, Epstein–Barr virus, mycophenolate mofetil, diffuse large B-cell lymphoma

## Abstract

Epstein–Barr virus (EBV)-positive primary central nervous system lymphoma (PCNSL) is a rare entity typically associated with profound immunosuppression, most commonly in transplant recipients or individuals with HIV. We report a case of EBV-positive PCNSL arising in a 75-year-old male with myasthenia gravis receiving chronic mycophenolate mofetil (MMF) therapy outside the transplant setting. The patient presented with progressive neurological deficits, and brain magnetic resonance imaging demonstrated multiple enhancing lesions. Stereotactic biopsy revealed diffuse large B-cell lymphoma of non–germinal center subtype with immunoblastic features and EBV-encoded RNA (EBER) positivity, confirming EBV-positive PCNSL. MMF was discontinued, and the patient was treated with rituximab and high-dose methotrexate, resulting in stable disease. This case highlights that prolonged MMF therapy may confer sufficient immunosuppression to permit EBV-driven lymphoproliferative disease even in non-transplant patients. Early recognition, withdrawal of immunosuppression, and initiation of methotrexate-based chemotherapy can lead to favorable outcomes.

## 1. Introduction

Primary central nervous system lymphoma (PCNSL) is an uncommon and aggressive extranodal non-Hodgkin lymphoma restricted to the brain, leptomeninges, spinal cord, or eyes [1]. It accounts for approximately 2% of all intracranial neoplasms and 4–6% of all non-Hodgkin lymphomas [2]. The disease most often presents as diffuse large B-cell lymphoma (DLBCL) of the nongerminal center (non-GC) B-cell type [1]. Although the majority of PCNSL cases in immunocompetent individuals are Epstein–Barr virus (EBV)-negative, a small subset (<5%) demonstrate EBV positivity [3]. In contrast, EBV is detected in nearly all PCNSL cases arising in the context of immunosuppression, such as in patients with HIV/AIDS, post-transplant immunosuppression, or iatrogenic immune deficiency [4].

EBV is a ubiquitous gammaherpesvirus that infects more than 90% of the adult population worldwide and persists lifelong in memory B-cells [5]. In immunocompetent hosts, cytotoxic CD8^+^ T-cell surveillance prevents uncontrolled proliferation of EBV-infected B-cells. However, in immunosuppressed individuals, impaired T-cell function allows EBV-driven transformation of infected B-cells and subsequent lymphomagenesis [6]. EBV-positive and EBV-negative PCNSL represent biologically distinct entities despite overlapping clinical presentations. EBV-negative PCNSL typically harbors mutations in genes such as MYD88, PIM1, and CD79B, reflecting activation of the NF-κB and B-cell receptor signaling pathways [7,8]. In contrast, EBV-positive PCNSL exhibits a lower mutational burden [9] and an absence of these canonical BCR/NF-κB driver mutations [8,10].

When mutations do occur in EBV-positive PCNSL, they most frequently involve SOCS1, leading to JAK-STAT pathway disinhibition, or gain-of-function mutations in NOTCH (1/3/4), with these two programs being mutually exclusive [10]. Transcriptomic and epigenomic profiling confirms that EBV-positive and EBV-negative PCNSL are distinct entities, with EBV-positive tumors characterized by global promoter hypomethylation and upregulated IL-10–JAK-STAT, NOTCH and viral life-cycle signaling, as opposed to the BCR/WNT-β-catenin-dominant program of EBV-negative disease [11]. Finally, the tumor microenvironment (TME) of EBV-positive PCNSL is consistently tolerogenic, with elevated M2 (CD163^+^) macrophage infiltration, immune checkpoint upregulation (PD-L1, LAG-3, TIM-3), and T-regulatory (FOXP3^+^) cell enrichment. Importantly, retention of HLA class I/II and intact antigen-presentation machinery in these tumors suggests immune evasion is achieved through active immunosuppression rather than antigen loss [8,10,12].

Here we present a case of EBV-positive PCNSL occurring in an elderly man receiving long-term mycophenolate mofetil (MMF) therapy for myasthenia gravis. This case underscores the complex interplay between viral oncogenesis and pharmacologic immunosuppression when utilizing a medication that is not typically thought of as immunosuppressive.

## 2. Case Presentation

A 75-year-old man with a history of myasthenia gravis, treated with mycophenolate mofetil (MMF) 1000 mg twice daily since 2018 and no other immunosuppressive agents, presented in 2021 with difficulty performing learned tasks, including parking his car and completing simple manual activities. These symptoms raised concern for higher-order motor dysfunction rather than a primary fine motor deficit. On examination, he was alert and fully oriented, with intact speech, mood, and affect. Cranial nerve testing was unremarkable except for bilateral hearing loss. Motor strength and tone were normal in all extremities, though a subtle right upper extremity drift was observed. Sensory, reflex, and coordination testing were normal. No clear focal deficits explaining his functional complaints were identified on examination.

**Figure 1:**
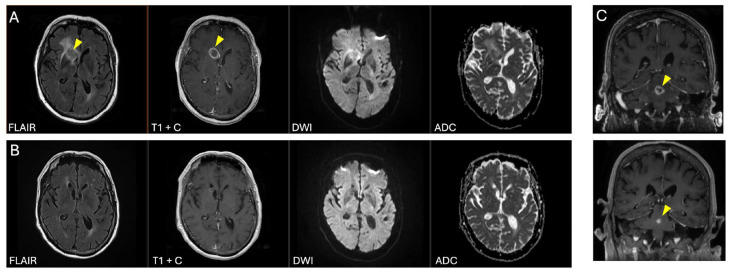
Axial and coronal MRI images demonstrating basal ganglia and pontine masses at sequential timepoints. (**A**) Axial images at initial presentation and (**B**) at surveillance entry showcasing basal ganglia mass (arrowhead). (**C**, **top**) Coronal MRI image at the time of mixed treatment response and (**C**, **bottom**) at surveillance entry with the pontine mass indicated (arrowhead). No diffusion restriction or microbleeds affected image quality. Abbreviations: FLAIR, Fluid-Attenuated Inversion Recovery; T1 + C, T1 with gadolinium contrast enhancement; DWI, Diffusion-Weighted Image; ADC, Apparent Diffusion Coefficient map.

**Figure 2:**
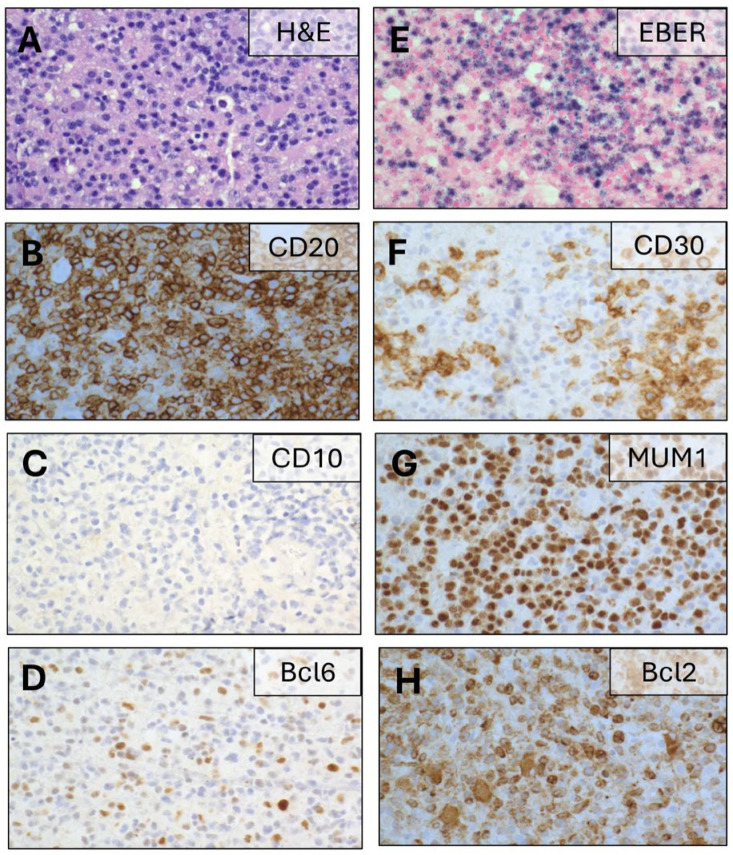
Immunohistochemistry of PCNSL specimen. (**A**) H&E staining showing a diffuse proliferation of large atypical lymphoid cells. (**B**) CD20 with strong diffuse membranous positivity. (**C**) Negative CD10 staining. (**D**) Scattered positive Bcl6 staining. (**E**) EBER-ISH with diffuse nuclear positivity confirming EBV infection. (**F**) Scattered positive CD30 staining. (**G**) MUM1 with strong diffuse nuclear positivity. (**H**) Bcl2 with diffuse cytoplasmic positivity. All stains were performed on formalin-fixed, paraffin-embedded tissue. Images taken at 40× magnification.

Routine laboratory studies showed a normal comprehensive metabolic panel and mild thrombocytopenia. Kidney function was preserved (eGFR 80 mL/min/1.73 m^2^). Magnetic resonance imaging (MRI) of the brain revealed multiple enhancing lesions, including a ring-enhancing mass in the right anterior basal ganglia (2.2 × 2.1 cm), a lesion in the left caudate (1.7 × 1.5 cm), and a smaller lesion in the dorsal pons (0.6 × 0.5 cm) (Figure 1A). High-dose corticosteroid therapy (dexamethasone 4 mg every 6 h) led to symptomatic improvement. A stereotactic biopsy of the basal ganglia lesion performed 11 days later confirmed primary diffuse large B-cell lymphoma (DLBCL) of the central nervous system. The patient remained on corticosteroids at the time of biopsy. Immunohistochemical staining demonstrated a MUM1^+^/BCL6^+^/CD10^−^ phenotype (Figure 2), consistent with a non-germinal center B-cell subtype by the Hans algorithm [13]. Scattered CD30-positive immunoblastic tumor cells were also identified. EBER in situ hybridization confirmed Epstein–Barr virus (EBV) positivity in the majority of B-cells. Clonal analysis was not performed. Additional immunohistochemical findings are summarized in Table 1. Peripheral blood EBV DNA quantification revealed 19,000 IU/mL (165 copies/μL; PCR log 4.3). Bone marrow evaluation, whole-body PET-CT, spinal MRI, slit-lamp examination, and testicular ultrasound showed no evidence of systemic disease, confirming EBV-positive primary CNS lymphoma (PCNSL).

Given the patient’s prolonged MMF exposure, immunosuppression was considered a contributing factor to EBV-driven lymphoproliferation. MMF was discontinued, and the patient was treated with rituximab (375 mg/m^2^ IV every 2 weeks for 5 doses), resulting in a partial response, with reduction in the basal ganglia lesion (2.0 × 1.9 cm) but progression of the pontine lesion (1.5 × 1.1 cm). A mixed response in a patient with ring-enhancing lesions raised concern for toxoplasmosis; therefore blood and CSF were tested. Though the Toxoplasma gondii PCR was negative, elevated IgG levels in both serum and CSF prompted a 14-day empiric course of leucovorin, pyrimethamine, and sulfadiazine with no radiographic improvement.

Treatment was subsequently escalated to rituximab plus high-dose methotrexate (3.5 g/m^2^ IV every 2 weeks for 5 doses), followed by maintenance rituximab. Serial monthly imaging demonstrated stable disease as defined by the 2005 international guidelines published by Abrey et al. [14], with interval reduction in both the basal ganglia and pontine lesion sizes, and a decrease in T2 signal abnormalities (Figure 1B,C). The patient entered surveillance in 2023. For management of myasthenia gravis, he was transitioned to prednisone 7.5 mg daily, along with maintenance rituximab infusions of 500 mg administered every 4 months, with intervals subsequently extended to 6 and then 9 months. Imaging intervals were progressively lengthened, with follow-up MRIs initially obtained every 2–3 months, then at 4, 6, and 9 months, and currently performed annually (every 12 months). Since entering surveillance, the patient has continued to demonstrate stable radiographic disease. Additionally, the patient’s myasthenia gravis remains well controlled, with fixed left ptosis as the only residual symptom. Peripheral blood EBV DNA quantification has not been performed during follow-up.

## 3. Discussion

Mycophenolate Mofetil and EBV-Driven PCNSL. This case illustrates EBV-positive PCNSL arising in the setting of chronic MMF therapy. MMF is metabolized to mycophenolic acid (MPA), an inosine monophosphate dehydrogenase (IMPDH) inhibitor that blocks de novo guanosine synthesis, thereby suppressing lymphocyte proliferation. T- and B-cells, which are heavily dependent on this pathway, are particularly affected. Despite its anti-proliferative effects on B-cells, MMF has paradoxically been associated with lymphomagenesis [15], and numerous case reports document PCNSL developing during MMF therapy [16,17,18,19,20,21,22,23,24], some of which explicitly confirm specimen EBV-positivity [16,19]. Notably, many of these reports involve patients with a pre-existing autoimmune condition, most frequently systemic lupus erythematosus (SLE) [16,17,20,21,22], for which MMF is a first-line agent. Though beyond the scope of this paper, it is worth mentioning that EBV has long been implicated in the pathogenesis of SLE [25,26,27], likely through molecular mimicry between viral proteins (i.e., EBNA2) and lupus autoantigens [28]. It is plausible that SLE patients harbor expanded reservoirs of EBV-infected B-cells, rendering them particularly vulnerable to EBV-driven lymphomagenesis upon pharmacologic immunosuppression, a consideration that should inform the clinical management of these patients.

Recently, the International Primary CNS Lymphoma Collaborative Group (IPCG) published a study on immunodeficiency-associated PCNSL [29], which provides important large-scale epidemiological context for our case report. Among 308 cases of immunodeficiency-associated primary CNS lymphoma (ID-PCNSL) from 23 centers in seven countries, mycophenolate mofetil was the most frequently used immunosuppressant (58.3%) in patients developing PCNSL in the setting of iatrogenic immunosuppression. This underscores the relevance of MMF as a risk substrate for EBV-driven CNS lymphomagenesis. Myasthenia gravis, the underlying condition in our patient, was also identified as one of the most common autoimmune diseases in this cohort, accounting for 9% of autoimmunity-related ID-PCNSL cases. Perhaps unsurprisingly, EBV was detected in 79.2% of all ID-PCNSL tumors. The combination of immune reconstitution and rituximab-methotrexate–based induction therapy was associated with superior response rates and prolonged progression-free survival, regardless of the underlying immunodeficiency etiology or tumoral EBV status.

Clinical Considerations in EBV Detection. At diagnosis, our patient exhibited EBV DNAemia with 165 copies/μL in blood. EBV status in clinical settings can be assessed through four main modalities: PCR, serology, heterophile antibody testing (Monospot), and EBER in situ hybridization (ISH). PCR-based detection quantifies viral DNA (commonly targeting EBNA1 or BamHIW repeat region), and while sensitive, is not specific since low-level EBV DNA can be found in healthy carriers [30,31,32]. For example, a positive PCR test may be a result of infected tumor cell lysis or by incidentally capturing an EBV-infected peripheral blood mononuclear cell (PBMC). EBV-infected PBMCs are common and can be detected in >60% of healthy blood donors [32], highlighting the risk of false positive PCR results.

Serologic assays detect antibodies to viral capsid antigen (VCA), Epstein–Barr nuclear antigen (EBNA) and, less commonly, early antigen (EA) [33]. A positive VCA IgM and VCA IgG with negative EBNA IgG indicates an acute infection; positive VCA IgG and EBNA IgG with negative VCA IgM suggests a past infection. The Monospot test detects transient heterophile IgM antibodies and is only useful for acute infection, not for latent or tumor-associated EBV detection [34]. EBER-ISH, which detects the abundant EBV-encoded microRNAs EBER1/2, remains the gold standard for confirming EBV presence within tumor tissue [35]. In our case report, the positive EBER-ISH definitively established the lymphoma as EBV-positive.

Radiographic and Pathologic Findings. Neuroimaging of this patient revealed multiple ring-enhancing lesions, a pattern often seen in EBV-positive PCNSL. This is in agreement with the IPCG study mentioned above, where EBV-positive tumors were significantly enriched for heterogeneous peripheral contrast enhancement and central necrosis on MRI [29]. However, this finding is not pathognomonic and overlaps with several differential diagnoses such as infection (i.e., toxoplasmosis, cryptococcus, and coccidiomycosis), metastatic disease, or demyelinating lesions [36]. Histopathologic evaluation demonstrated a non-germinal center B-cell (non-GCB) phenotype, consistent with an activated B-cell origin [37]. Non-GCB PCNSL typically arises from post-germinal center B-cells that have undergone antigen exposure and exhibit activation of pathways such as JAK/STAT and PD-L1–mediated immune evasion [7,38]. EBV-positive DLBCLs most often fall within the non-GCB category, whereas GCB EBV-positive DLBCL is less common and generally carries a more favorable prognosis [39]. CD30-positive immunoblastic morphology, as seen in this case, reflects differentiation toward plasmablasts or plasma cells, consistent with EBV’s propensity to establish latency in memory B-cells and propagate during plasmacytic differentiation [6,40].

Therapeutic Response. The treatment paradigms for EBV-positive vs. EBV-negative PCNSL are similar, with the exception that for EBV-positive PCNSL, immunosuppression is typically reversed if possible before initiation of treatment. The combination of rituximab and high-dose methotrexate (MTX) remains the cornerstone of PCNSL treatment, with whole brain radiation therapy (WBRT) and bruton tyrosine kinase (BTK) inhibitors typically reserved for refractory disease. Rituximab targets CD20^+^ B-cells through complement-dependent cytotoxicity, antibody-dependent T-cell-mediated cytotoxicity, and inhibition of B-cell proliferation [41]. In addition to depleting B-cells, rituximab may facilitate T-cell recognition of EBV-infected B-cells by increasing the pool of exogenous viral antigens generated during B-cell lysis [42]. Paradoxically, rituximab does not appear to cross the blood–brain barrier efficiently, making its full contribution to the treatment of PCNSL unclear [43]. Methotrexate is an antifolate agent that interferes with DNA and RNA synthesis. Interestingly, there is evidence that MTX can cause EBV lytic reactivation, thereby increasing tumor immunogenicity in EBV-positive tumors [44,45].

EBV-directed therapies. EBV-directed immunotherapies are a clinically underutilized strategy for treating patients with EBV-positive PCNSL but should be considered in the absence of a therapeutic response to the standard of care. As early as 1998, allogenic transfer of EBV-specific cytotoxic T lymphocytes (CTLs) had been shown to successfully prevent the development of and/or eliminate EBV-positive post-transplant lymphoproliferative disorder [46]. In more recent years, autogenic CTLs have been shown to successfully achieve complete response in patients with Hodgkin and non-Hodgkin lymphoma [47], though none of the patients included in this study had PCNSL. A timely review by Corey Smith and Rajiv Khanna discusses recent promising clinical data on adoptive transfer of virus-specific T-cells (VSTs), with an emphasis on the short-term and long-term effectiveness of EBV-directed VSTs in the management of EBV-positive lymphoma, though less effective in EBV-positive epithelial cancers [48]. At the time of this report, only one T-cell-based immunotherapy against EBV-positive post-transplant lymphoproliferative disease (PTLD) is approved and available in Europe: Tabelecleucel. Of note, in the United States, Tabelecleucel was granted priority review by the Food and Drug Administration in July 2025 with the plan for a final decision to be made in early 2026.

Beyond EBV-directed strategies, CD19-directed chimeric antigen receptor (CAR) T-cell therapy has emerged as a promising salvage option for relapsed/refractory PCNSL. CD19 is uniformly expressed on EBV-transformed B-cells and PCNSL tumor cells regardless of EBV status, providing a consistent therapeutic target. Intravenously administered CD19 CAR T-cells have been demonstrated to cross the blood–brain barrier and are detectable in cerebrospinal fluid [49], enabling direct intracranial antitumor activity. Early phase I/II data published in 2022 by Siddiqi et al. utilizing CD19-directed CAR T-cells in PCNSL demonstrated complete remission in 60% of treated patients with manageable toxicity profiles [50]. More recently, in 2024 an analysis of commercial CAR T (axicel or tisacel) in relapsed/refractory PCNSL demonstrated comparable 2-year overall survival and progression-free survival to patients with large B-cell lymphoma without CNS involvement [51]. Critically, in February 2026, the FDA approved an update to the axicabtagene ciloleucel (Yescarta) label, specifically removing the prior exclusion of patients with PCNSL, formally opening CAR T-cell therapy as a commercially available option in this setting. Given the tolerogenic, checkpoint-rich TME of EBV-positive PCNSL identified in molecular studies [8,10,12], rational combinations of CAR T-cell therapy with immune checkpoint inhibition (e.g., anti-PD-1) or JAK inhibitors (e.g., ruxolitinib for SOCS1-mutant disease) may further enhance antitumor activity and are an important area for future investigation.

## 4. Conclusions

This case underscores the importance of recognizing EBV-positive PCNSL as a distinct clinicopathologic entity that can arise in the setting of mycophenolate-induced immunosuppression. Clinicians should maintain a high index of suspicion for EBV-related lymphomas in patients presenting with CNS lesions while receiving MMF or similar agents. As immune-modulating therapies become increasingly prevalent, awareness of their oncogenic potential, particularly in the context of latent viral infections, remains critical. Standard-of-care therapy with rituximab and high-dose methotrexate can achieve durable disease control in EBV-positive PCNSL, as demonstrated in this case. Looking ahead, an expanding arsenal of targeted and cellular immunotherapies holds promise for patients with refractory or relapsed EBV-positive PCNSL.

## Figures and Tables

**Table 1:** Histologic summary of patient specimen.

Marker	Status
GFAP	Negative
CD20	Positive
OLIG2	Negative
CAM 5.2	Negative
CD45	Positive
MART1/HMB45	Negative
CD10	Negative
CD3	Stains few scattered small T-cells
CD5	Stains few scattered small T-cells
CD30	Stains several groups of medium-sized cells, about 20% of all cells
MUM-1	Positive
Ki-67	Proliferation index of about 40%
BCL-2	Moderately positive
BCL-6	A minority of B-cells stain positive
CMYC	Negative, less than 5% nuclei stain
EBER ISH	Positive

## Data Availability

The original contributions presented in this study are included in the article. Further inquiries can be directed to the corresponding author. Additional data regarding this case study will be provided upon reasonable request in accordance with DUHS IRB policies.
